# Wildlife, human and environmental costs of using lead ammunition: An economic review and analysis

**DOI:** 10.1007/s13280-019-01157-2

**Published:** 2019-03-16

**Authors:** Deborah J. Pain, Ian Dickie, Rhys E. Green, Niels Kanstrup, Ruth Cromie

**Affiliations:** 1 Department of Zoology, University of Cambridge, David Attenborough Building, Pembroke Street, Cambridge, CB2 3QZ UK; 2 Wildfowl & Wetlands Trust, Slimbridge, Gloucestershire GL2 7BT UK; 3 eftec - economics for the environment, 4 City Road, London, EC1Y 2AA UK; 4 Department of Bioscience, Aarhus University, Grenåvej 14, 8410 Rønde, Denmark

**Keywords:** Birds, Bullets, Evaluation, Financial, Gunshot, Society

## Abstract

A proposed European Union (EU)-wide restriction on the use of lead gunshot for shooting in and over wetlands estimated that the societal benefits of a restriction outweighed costs, despite few identified benefits being quantified economically. A subsequent Annex XV Investigation Report on the evidence of impacts of lead ammunition in terrestrial environments concluded that additional measures to control its use are warranted, although to date this has not been further evaluated. To help inform this process, we review the literature and undertake new analyses to estimate the costs of continued use of lead ammunition associated with impacts on wildlife, people and the environment. We estimate minimum annual direct costs across the EU and Europe of c. €383 million–€960 million and €444 million–€1.3 thousand million respectively. The value that society places on being able to avoid these losses, estimated using a ‘willingness to pay’ approach, was c. €2.2 thousand million for wildfowl alone. Our estimated costs of the continued use of lead ammunition across the EU appear to be considerably greater than the likely costs of switching to non‐toxic alternative ammunition types, although these have not been formally estimated in full.

## Introduction

Due to the high toxicity of lead and the public and environmental health problems it causes, most releases of lead into the environment are strictly regulated in Europe (e.g. see AMEC 2012). However, shooting continues to release tens of thousands of tonnes of lead ammunition (gunshot and bullets) into the European environment annually, contaminating soil and water and putting at risk the health of wild birds that ingest spent ammunition directly, and both wildlife and people that eat lead ammunition or fragments of it in their food. While limited regulations exist requiring the replacement of lead with non-toxic ammunition in some parts of the world and for certain types of shooting, these do not adequately control the risks (see Green and Pain 2012, 2015; Pain et al. 2015, 2019). In Europe, a few countries banned the use of lead gunshot decades ago (e.g. Denmark and the Netherlands), but in most EU Member States, controls are partial, piecemeal, and not always complied with (Cromie et al. 2015). Based on the overwhelming evidence of the toxic effects of lead from ammunition in wildlife and the risks to human health, scientists (Bellinger et al. 2013; Group of Scientists 2014) and Multilateral Environmental Agreements (AEWA 1999; CMS 2014; IUCN 2016), have called for the replacement of lead ammunition with non-toxic alternatives. The European Commission requested the European Chemicals Agency (ECHA) to prepare an Annex XV report proposing a restriction on lead gunshot in wetlands under the EU REACH (Registration, Evaluation, Authorisation and Restriction of Chemicals) Regulation. The report was reviewed and adopted by ECHA’s expert scientific committees (including the Committee for Socio-Economic Analysis) in June 2018, prior to being considered by the REACH Committee (comprising Member State representatives) before adoption into law.^1^ Concurrently, ECHA published an Annex XV Investigation Report on the evidence of impacts of lead ammunition in terrestrial environments (ECHA 2018a) which recommends that additional measures are needed to regulate the use of lead ammunition in terrestrial environments and lead fishing weights. While the costs of replacing lead gunshot with non-toxic alternatives have been widely considered (e.g. AMEC 2012), there have been only a few incomplete attempts at quantifying the costs of continued use of lead ammunition (e.g. Andreotti et al. 2018). Hence, the net costs/benefits of restriction remain uncertain.

In this paper, we review cost estimates in the peer-reviewed and grey literature, including consultation responses to the ECHA restriction proposal (ECHA 2017b, 2018c) and add new analyses of the costs to society of continued use of lead ammunition. New analyses include replacement costs of four species of raptors in the EU and Europe, replacement costs of several species of terrestrial birds in the UK, and costs of potential reductions in IQ in children in the EU, resulting from frequent consumption of wild game shot with lead. We also highlight other as yet unquantified costs. This paper is not exhaustive but aims to give an indication of the types and magnitudes of some of the main costs of continued use of lead ammunition to society.

## Assessment of costs associated with the use of lead-based ammunition

### Costs associated with impacts on wildlife

Lead poisoning from ammunition sources affects a wide range of different species, but most research has involved birds. This has been reviewed by numerous authors, updated by Pain et al. (2019). Wildfowl (ducks, geese and swans) ingest spent lead gunshot while feeding, mistakenly for food items or the grit that they deliberately ingest and retain in a muscular part of their stomach, the gizzard, to help break down their food. Other birds that directly ingest spent gunshot include other waterbird species (e.g. cranes and flamingos) and terrestrial birds including gamebirds like partridges and pheasants, pigeons and doves. Another route of exposure occurs in predatory of scavenging birds, whose food includes species that are shot as pests or for food or sport. Raptors and scavenging birds can eat shot, bullets or fragments thereof in shot animals that have been wounded and survived (and thus may be more vulnerable to being taken by a predator) or unretrieved carcasses. As a common practice, parts of carcasses of large game animals, like deer viscera, are removed and left in the countryside when the animal is retrieved. These may contain fragments of ammunition. Recent evidence indicates that mammalian predators and scavengers may similarly be exposed to dietary lead derived from ammunition (reviewed in Pain et al. 2019), but this has not been widely investigated. Finally, some birds with lead ammunition shot into their bodies are not killed by it immediately, but their subsequent welfare and survival may be adversely affected. In addition to the wildlife killed directly by lead poisoning, several times more animals suffer welfare effects from sublethal poisoning (Andreotti et al. 2018) and may have increased susceptibility to other diseases or accidents (Kelly and Kelly 2005; Ecke et al. 2017).

The costs to society of sublethal poisoning and mortality of wildlife are difficult to evaluate, but the question can be approached in a variety of ways. These ways include estimating the

- Costs of *replacing* birds that have died. This could be through captive breeding and release or other means of increasing the populations.
- Costs of *treating* poisoned birds.
- Costs of *losing the services* provided by the wildlife, including tourism, hunting for food or sport and improvement of environmental health.
- *Willingness of society to pay* to avoid these impacts—a way of estimating the value of wildlife to people.

These approaches are described below.

#### Replacement costs

(i) *Wildfowl* For 16 of species of wildfowl for which sufficient information was available, Andreotti et al. (2018) estimated that about 700 000 individuals die from acute lead poisoning annually in the EU (6.1% of the wintering population) and one million across Europe (7.0%). Three times more birds were estimated to suffer sublethal effects. These authors estimated the economic loss of the acute mortality by calculating the replacement costs through buying and releasing captive-bred birds, taking account of the high mortality rate of captive birds (72.7%) in the months following release into the wild. This was estimated at an annual cost of €105 million in the EU and €142 million across Europe. These figures are for 16 species only and do not include species for which there were insufficient data. Deaths caused indirectly by lead poisoning and effects on reproduction were excluded and if included would increase the estimated losses.

It is notable that of the 150 migratory waterbird species listed under the African-Eurasian Waterbird Agreement (AEWA) which regularly occur within the EU, two thirds (100 species) are considered to be vulnerable to lead poisoning from spent lead shot based upon research and surveillance (where available) and knowledge of feeding behaviour and habits (AEWA 2017). Lead poisoning is a threat to 23 wildfowl species with unfavourable conservation status for which single species action plans have been written (Kanstrup et al. 2018). Replacement costs for all affected waterbird species would therefore be considerably higher than these estimates.

(ii) *Terrestrial gamebirds* Although terrestrial gamebirds ingest shot, suffer sublethal effects and can die of lead poisoning, it is more difficult to estimate numbers that die of lead poisoning each year. This is primarily because little information exists on their sensitivity to lead poisoning and fewer studies have been conducted on them. However, of these, several suggest that some terrestrial gamebirds may be less sensitive to the effects of lead poisoning than wildfowl (Gasparik et al. 2012; Runia and Solem 2017). In the UK, sufficient information exists to calculate, in broad terms, potential gamebird mortality from lead poisoning and how much it would cost to replace birds lost, based upon levels of shot ingestion and production costs of reared and released pheasants (*Phasianus colchicus*). Table 1 outlines our calculation of replacement costs. Our estimates of mortality are based on a method used by Bellrose (1959) to estimate lead poisoning mortality in wildfowl. Due to the uncertainties mentioned above, and because some gamebirds that might otherwise die from lead poisoning are shot before they can do so, we have been conservative in our terrestrial gamebird mortality estimates in several ways (Table 1). For example, we have assumed that gamebirds ingest only one shot (while pheasants are known to frequently ingest multiple shot, for example,. Runia and Solem 2016) and that mortality from lead poisoning following shot ingestion is an arbitrary 50% of that estimated for mallards (*Anas platyrhynchos*). Consequently, while terrestrial bird mortality could still be smaller than our estimate, it is perhaps more likely to be greater. In contrast, a bias that would result in our estimate of lead poisoning mortality being too high results from the substantial non-shooting mortality of captive-bred birds in the first few weeks post-release (Madden et al. 2018); this would result in many fewer birds surviving to be exposed to lead poisoning. Ideally, we would wish to estimate lead poisoning mortality based upon numbers of birds that survive for different periods post release, and would also correct for the fact that not all pheasants are released simultaneously. These biases in both directions highlight that our estimate should be considered only as a very broad indicator of the possible magnitude of lead-poisoning-related costs. We estimate that, in the UK, 232 402 pheasants and red-legged partridges (*Alectoris rufa*) may die as a direct result of lead poisoning each year. We multiplied this by the cost of producing and releasing a pheasant to arrive at an estimated annual replacement cost of over €3 million in the UK (Table 1). This is a broad approximate estimate rather than a precise estimate and does not include the contribution of sublethal lead poisoning to increased levels of mortality from other causes. We have been unable to find EU-wide figures for numbers of terrestrial gamebirds released and rearing costs.

**Table 1:** Estimated replacement costs of common pheasants and red-legged partridges affected by lead shot from ammunition sources in the UK

	% hunter shot birds with ingested shot	Number of shot ingested^a^	Hunting bias correction^b^	Percentage with ingested gunshot after hunting bias correction	Percentage with ingested shot corrected for turnover^c^	Increase in annual mortality for birds with ingested shot^d^	Percentage of population estimated as dying^e^	Population^f^	Number of birds estimated as dying	Cost of replacement	References
Pheasant	3.0	1	1.5	2.00	12.4	0.045	0.558	37 800 000	210 924	€3.132 million	Butler et al. (2005)
Red-legged partridge	1.4	1	1.5	0.93	7.2	0.045	0.323	6 665 000	21 478	€0.319 million	Butler (2005)
Total									232 402	€3.451 million	

Assumptions:

^a^As we do not know how many shot are ingested, we assume that only one shot is ingested

^b^We assume that the increased likelihood of a hunter killing a terrestrial gamebird that has ingested a lead gunshot is the same as a mallard that has ingested a lead gunshot. If terrestrial gamebirds are less sensitive to the effects of lead, then hunters will be less likely to kill a pheasant that has ingested lead

^c^We use a hunting season of 124 days in the UK for pheasant (1 Oct–1 Feb) and 154 days for red-legged partridge (1 September–1 February) and a turnover rate of shot in the gizzard of 20 days—i.e. 124/20 = 6.2 for pheasant; 154/20 = 7.7 for red-legged partridge)

^d^Due to the possibility of decreased sensitivity of terrestrial gamebirds to lead-shot impacts, we have used an arbitrary increase in annual mortality caused by lead-shot ingestion of 50% of that calculated for mallard (Bellrose 1959)

^e^Percentage with ingested shot corrected for hunting bias and turnover multiplied by increase in annual mortality

^f^Assumes that 35 million pheasants and 6.5 million red-legged partridges are released each year (PACEC 2006), although this is likely to be an underestimate as numbers of released birds are reported to have increased (Aebischer 2013). We added on breeding numbers from Musgrove et al. (2013) assuming a ratio of male to breeding female pheasants of 1:4.6 and that each red-legged partridge territory equalled 2 birds. We multiplied by the production cost of each pheasant released of €14.85 (£12.55; Savills 2017), and assumed that this was similar for red-legged partridges

(iii) *Predatory and scavenging raptors* Table 2 gives our minimum estimated annual costs of replacing individuals of four of the 16 species of raptor (Pain et al. 2019—Table 2) known to be susceptible to lead poisoning in Europe. While many more raptor species die of lead poisoning, insufficient data were available to estimate replacement costs in other species. Estimates of numbers of individuals that die from lead poisoning annually were based on EU or European population size of breeding pairs, mean annual adult survival, and the percentage of mortality estimated to be from lead poisoning taken from relevant studies. Replacement costs were based on an analysis of the costs of releasing immature raptors to the wild for reintroduction or population supplementation (Ferrer et al. 2017) and range from what are considered to be very cost effective (supplementary feeding and translocation) and more costly (breeding and reintroduction) methods. These figures were then scaled to account for the proportion of fledgling birds that survive to reproductive age.

**Table 2:** Estimated replacement costs of selected raptor species killed by lead poisoning from ammunition sources in the European Union and throughout Europe

Species	Population (pairs) EU; Europe^a^	Annual adult survival^b^	Numbers of adults (individuals) estimated to die annually EU; Europe	Percentage of mortality estimated from lead poisoning^c^	Numbers of adults estimated to die annually from lead poisoning EU; Europe	Replacement cost (€48 108 per adult) EU; Europe	Replacement cost (€661 284 per adult) EU; Europe	References
White-tailed eagle	4202; 10 650	0.90–0.95	420–840; 1066–2130	22	92–184; 234–468	€4.4–8.9 million; €11.3–22.5 million	€61.1–122.3 million; €154.9–309.9 million	Isomursu et al. (2018) [Finland], Krone et al. (2009) [Germany], Nadjafzadeh et al. (2013) [Germany], Helander et al. (2009) [Sweden]
Golden eagle	5300; 10 800	0.87	1378; 2808	5–10	69–138; 140–281	€3.3–6.6 million; €6.8–13.5 million	€45.6–91.1 million; €92.8–185.7 million	Ganz et al. (2018) [Swiss Alps], Ecke et al. (2017) [Sweden], Russel and Franson (2014) [USA], Langner et al. (2015) [USA]
Griffon vulture	32 350; 33 400	0.97 (released—long-term estimate)	1941; 2004	2.5	49; 50	€2.3 million; €2.4 million	€32.1 million; €33.1 million	Berny et al. (2015) [French Pyrenees]
Red kite *Milvus milvus*	27 950; 29 300	0.92 (3rd year in absence of illegal killing)	4472; 4688	7.15	320; 335	€15.4 million; €16.1 million	€211.4 million; €221.7 million	Molenaar et al. (2017) [England], Berny et al. (2015) [French Pyrenees]
Total—EU; Europe					530–691; 760–1135	€25.4–33.2 million; €36.6–54.5 million	€350.2–456.9 million; €502.5–750.4 million	

^a^Adult populations (pairs) from BirdLife International (2015). The European Red List of Birds Supplementary Material. Adults are given as no or limited information is available for immature birds. However, total population sizes, estimates of numbers of birds that will die of lead poisoning, and replacement costs would be considerably larger

^b^Recent survival estimates used where possible. Where possible, these are for stable or increasing populations. This will result in an underestimate of mortality and costs

^c^For white-tailed eagles, 22% of 740 dead birds is the average from four large studies in Finland, Germany and Sweden. For golden eagles, 9% of 55 birds in the Swiss Alps had liver Pb > 6 mg/kg (up to 80 mg/kg), and 30% of 46 birds had bone Pb > 20 mg/kg. We use a range of 5–10%, as has been found in the USA. For griffon vultures, a recent surveillance programme (French Pyrenees) reported 2.5% of 119 dead birds as lead poisoned. For red kites, 5.5% of 110 red kites (England) and 8.8% of 34 dead birds in the French Pyrenees died from lead poisoning (midpoint of 7.15%). Both immature and adult birds are included in these estimates

^d^These are minimum costs because of the assumptions above^a,b^. Costs are based on an analysis of what are considered to be cost-effective (supplementary feeding and translocation) and more costly (breeding and reintroduction) methods of replacing birds lost. These are based on Ferrer et al. (2017) who calculated that for a standard reintroduction programme of bearded vultures, releasing 10 young per year during 10 years, each one of the released young bred in captivity costs around €146 805 compared with €10 680 for each young bird that originated from a food-supplemented wild population. The activities undertaken to reintroduce or supplement populations of many raptor species, and thus their costs, are broadly similar. The proportion of fledgling birds that survive to reproductive age has been shown to be relatively constant in wild bird populations (Ricklefs 2000; see also Fig. 7.1 of Green 2002), with a mean value of 0.222. To obtain the cost of replacing one dead adult, we therefore divided the cost of producing an immature by 0.222 to give replacement costs per adult of €661 284 and €48 108, respectively

We estimate annual replacement costs to be €25–457 million in the EU and €37–750 million in Europe for these four species alone. The wide range takes into account different approaches to replacing wild birds, from supplementary feeding and translocation (€48 108 per adult bird) to captive breeding and reintroduction (€661 284 per adult bird). Minimum costs are likely to be between the middle and upper ends of this range because even the costs of the generally cheaper population supplementation method can fall in the middle of this range, as illustrated by a recent golden eagle (*Aquila chrysaetos*) population supplementation project in Scotland (Pinkstone 2017). Notably, it is not always possible to supplementary feed wild birds to increase productivity and translocate additional young.

Our estimates should also be considered as minima because immature birds as well as adults die from lead poisoning and survival estimates used tended to be for stable/increasing populations and may underestimate overall mortality.

#### Treatment costs

An alternative to replacing wildfowl lost to lead poisoning would be to find and treat all poisoned birds. For wildfowl, treatment costs would be approximately €1 000^2^ a bird covering an anaesthetic and X-ray, blood test for diagnosis, five days of hospitalisation with lead-chelation therapy and one more accompanying blood test. This is likely to be a minimum level of treatment. Treating the 1 million wildfowl estimated to die in Europe each year would therefore cost c. €1 thousand million a year and with the additional 3 million wildfowl that suffer sublethal effects would cost €4 thousand million a year. However, finding, catching and treating all such birds is not a practical proposition even were financial resources available as it would only be possible to find a small proportion of poisoned birds in a condition that would allow for their treatment prior to death. It is difficult to estimate with any precision the proportion of birds potentially treatable, so in order to generate indicative costs, we assume here that 1% of all lead-poisoned birds could be treated. Assuming 1% of the estimated 1 million wildfowl dying every winter in Europe could be treated, this equates to avoided costs of €10 million per year. Treatment costs for 1% of the million birds that die plus 1% of the 3 million additional birds estimated to suffer welfare effects from lead poisoning would be €40 million per year (€28 million a year for wildfowl in the EU). Treating and thus potentially avoiding the deaths of 1% of all poisoned wildfowl would be largely additional to replacement costs as welfare organisations would treat sick and dying birds irrespective of replacement.

These figures are substantial underestimates as the costs of finding sick birds are likely to be greater than treatment costs and these have not been included. We have not estimated costs of treating raptors, other scavengers or terrestrial birds.

#### Costs of services lost

Wild birds provide a large number of services to society, some of which are outlined below.

(i) *Birdwatching* Many people across Europe enjoy birdwatching. In the UK alone, six million people were reported to enjoy birdwatching every couple of weeks (Kellaway 2009). People benefit physically and mentally from walking in greenspaces of high natural value and from exposure to birds and other nature (e.g. Barton et al. 2009; Cox et al. 2017, 2018), and many industries benefit economically from birdwatching including optics (binoculars, telescopes and cameras), publishing, bird food, tourism and associated industries. While it is difficult to quantify the economic impact on human health and wellbeing of the reduction in quality of the natural environment caused by the avoidable loss of birds due to lead poisoning, other economic values are more readily quantified. For example, white-tailed eagles (*Haliaeetus albicilla*) were driven to extinction in Scotland at the beginning of the twentieth century largely as a result of persecution, and were first reintroduced to Scotland in 1975. Surveys on the Scottish Isle of Mull conducted in 2010 found that up to £5 million (€5.9 million^3^) of tourist spend was attracted every year by the (at the time) 14 pairs of white-tailed eagles that had recolonised the island; 110 jobs were supported by this spend each year, and £1.4 million (€1.65 million) of local income was supported each year (Molloy 2011). In many parts of the white-tailed eagle’s range, lead poisoning is an important mortality factor (Table 2). Applying the average figures for annual adult survival and loss to lead poisoning for white-tailed eagles across Europe (Table 2) would give an estimated loss to the local economy of the Isle of Mull, with a population of just 3000 people, of £82 500 (€97 350) (annual survival of 0.925, 22% of mortality from lead poisoning equates to 0.462 adult breeding birds lost to lead poisoning annually, with a value of £5 million (€5.9 million) for 28 adult breeding birds). This figure is simply illustrative, as average survival and lead-poisoning figures from across Europe do not necessarily apply to the eagle population on the Isle of Mull, but gives an indication of the potential value of even small numbers of raptors to local communities.

Specific birdwatching opportunities and general interest in birds also generate revenue. Examples are goose-watching in Scotland, estimated at £1.5 million (€1.77 million) a year more than 20 years ago (Rayment et al. 1998). More generally, in 2015, there were around 2.2 million individuals and family members of BirdLife partner-organizations in the EU, Norway, Iceland and Liechtenstein (BirdLife International 2018). Members of the 10 EU BirdLife partner-organisations with the most members spend a total of €126 million a year in fees (BirdLife International 2018). In addition, there are many other conservation organisations across Europe members of which have an interest in birds. While it is not easy to use these figures to ascribe a value to the loss of birds to lead poisoning, it highlights some of the value that people place upon birds—further reinforced by a ‘willingness to pay’ (WTP) study—illustrated below.

(ii) *Hunting for sport or food* Game species of wetland and terrestrial birds provide leisure hunting opportunities and harvest opportunities for meat or for feathers. Andreotti et al. (2018) estimated the annual cost of the opportunities lost for hunting caused by mortality in the 16 wildfowl species to be €129 million in the EU and €185 million across Europe. In the 2017/18 season, about 38% of pheasants and red-legged partridges released in the UK were shot and the average income per bird shot was c. £36 (€42.5—Savills 2017). Therefore, income lost in the UK as a result of lead-poisoning deaths of an estimated 232 402 pheasants and partridges (see above) would be an estimated £3.18 million (€3.75 million).

(iii) *Environmental and human health* Wild birds support environmental health in variety of ways, a clear example being that of scavenging raptors, which remove potentially biohazardous material from our environment (summarised by Birdlife International 2018). Vultures, as scavengers, are particularly vulnerable to the ingestion of lead from ammunition in the carcasses of dead large game animals, and losing their services comes at a cost. As an example, following an outbreak of bovine spongiform encephalopathy (BSE) in 2001 and the detection of Creutzfeldt–Jakob disease in humans, sanitary legislation (Regulation EC 1774/2002) was passed in the EU requiring that domestic animal carcasses be collected from farms and transformed for use for industrial purposes or destroyed in authorised plants. This reduced the food supply for the vultures that had traditionally relied in part on the flesh of domestic livestock for their food, consequently providing an important environmental health service. Morales-Reyes et al. (2015) estimated that in Spain (which holds 90% of European vultures—BirdLife International 2015), carcass collection and transport to processing plants resulted in additional emissions of 77 344 metric tons of CO_2_ eq. to the atmosphere per year, plus payments by farmers and regional/national administrations ca. $50 million (€44 million)^4^ to insurance companies for livestock carcass removal and processing in 2012. Although new legislation (Regulation EC 142/2011) in 2011 allowed for disposal of carcasses in set areas where vultures could feed, this analysis illustrates the economic value of the disposal service and avoided CO_2_ emissions provided by vultures. In France, it is estimated that livestock carcass removal by 900 pairs of griffon vultures (*Gyps fulvus*) saves the public purse €440 000 a year (Orabi 2011). In India, massive population declines in three species of *Gyps* vulture were estimated to have associated human health costs (resulting from increases in feral dogs, dog bites and human rabies cases) of US$ 34 thousand million (€29.92 thousand million) between 1993 and 2006 (> $2 thousand million—€1.76 thousand million—a year; Markandya et al. 2008).

(iv) *Other Services* Many species help with the dispersal of plants and lower organisms supporting ecosystem functioning. Waterbirds alone provide a range of key services via their roles in many aquatic ecosystems (Green and Elmberg 2014). These include as predators (including of ‘pest’ species), herbivores and vectors of seeds, invertebrates and nutrients. Many species can be effective sentinels of potential disease outbreaks and bioindicators of ecological conditions. While we have not attempted to estimate the value of the services lost as a result of lead poisoning, Green and Elmer (2014) suggest some methodologies for calculating value of waterbirds.

#### Willingness to pay (WTP)

It is estimated that in the EU, about 700 000 wildfowl die every winter as a direct result of lead poisoning (Andreotti et al. 2018), representing 6.1% of the wintering population. This is a minimum as additional birds that suffer sublethal poisoning are likely to die from other causes, exacerbated by the sublethal poisoning. A WTP study in Scotland found that on average, people were willing to pay an estimated £10.99 (€16.50 in 2017 prices)^5^ per household per year for avoided losses of 10% in all goose species (Hanley et al. 2001).

In the absence of better valuation evidence, the Scottish value can be extrapolated to the number of EU households in 2017 (~ 221 million—Eurostat 2018),^6^ calibrated for the avoided losses of 6.1% of all species of wildfowl in the EU (see WWT 2018).

There is uncertainty involved in applying WTP values from one country and environmental ‘good’ to another. Value transfer guidelines (eftec 2009) have been considered regarding the calculation of these values, and the key criteria to be considered in assessing the suitability of a study good (the geese valued in Hanley et al. 2001) to the policy good (the impacts on wildfowl of lead shot) are outlined in Table 3. As shown, the differences in the species being addressed within the study good and policy good, and the location and affected populations, are key areas of uncertainty for this value transfer. The indicative value in the EU for the avoided losses in wildfowl populations obtained from this value transfer may be €2.2 thousand million per year. This transfer from Scotland to the rest of the EU, and the fact that the WTP study only valued geese populations, introduces significant uncertainty. It is possible that values are significantly under- or over-estimated and the estimate should be regarded as a broad indicative value. Further adjustments to this transfer (e.g. to account for different rates of environmental NGO membership) could be made, but doing so would not counter the main sources of uncertainty—around whether households in the rest of the EU hold similar views to households in Scotland, and whether public preferences have changed between 2001 and 2018. Despite this uncertainty, this value transfer illustrates that households in the EU are likely to hold a very significant positive value for avoiding wildfowl deaths caused by lead poisoning from gunshot ingestion.

**Table 3:** Value transfer—comparing policy good context and study good context(s)

Selection criteria	Hanley et al. (2001)	ECHA policy site and good	Conclusion
I. Similarity of the study good and policy good	Avoided loss of goose populations	Avoided loss in all species of wildfowl populations (due to decrease in lead poisoning mortality)	Similar
II. Similarity of the change in provision of the study good and policy good	10% population decrease	6.1% population decrease	Adjust values for different % level of loss
III. Similarity of the sites where the study good and policy good are found	Goose habitats across Scotland, UK	Wildfowl habitats across EU Member States	Similar
IV. Similarity of the affected human populations	Scotland population (households) 2001	EU population (households) 2017	Sites are similar, but adjust for number of households, and disposable income per household
V. Similarity of the number and quality of substitutes for the study good and policy good	Substitutes (other wildfowl and bird species are conserved)	Some substitutes (other bird species are conserved)	Similar
VI. Similarity of the study good and policy good market constructs	Public good	Public good	Similar
VII. Study quality	A robust study with a full account of validity and potential biases in estimates	N/A	Good quality

The costs estimated in this section deal only with those relating to wildfowl. The WTP value of avoiding declines of other species would be considerable. People also appear willing to pay a considerable amount to avoid declines of threatened species (Hanley et al. 2001). Many species considered to be regionally threatened in Europe and the EU are at risk from lead poisoning (e.g. Egyptian vulture (*Neophron percnopterus*), bearded vulture (*Gypaetus barbatus*), Spanish imperial eagle (*Aquila adalberti*), common pochard (*Aythya ferina*) and many other wildfowl species (see Pain et al. 2015, 2019; and Leronymidou et al. 2015 for species status). Avoiding lead poisoning in these species is certainly desirable from a population perspective, and the costs of their recovery would be substantial and long term.

### Cost associated with impacts on people

Fragments of lead derived from the ammunition used to kill game birds and mammals are often present in their edible tissues and are a potentially significant source of dietary exposure to bioavailable lead in groups of people who frequently consume the meat of game animals (EFSA 2010; Pain et al. 2010; Green and Pain 2012). The Panel on Contaminants in the Food Chain (CONTAM Panel) of the European Food Safety Authority (EFSA) produced a scientific opinion on lead in food (EFSA 2010) at the request of the European Commission, and for their risk assessment identified critical effects in humans as being developmental neurotoxicity in young children and cardiovascular effects and nephrotoxicity in adults. Their reference points for characterising increased risk from dietary lead were Benchmark Dose Limits (BMDLs), being the 95th percentile lower confidence limit of the Benchmark Dose—BMD—of extra risk derived from blood lead levels in μg/L^−1^. There is evidence that the developing brains of children are especially susceptible to the effects of chronic lead exposure, even at low concentrations (Lanphear et al. 2005; Budtz-Jørgensen 2010; EFSA 2010). The EFSA CONTAM Panel concluded from their risk assessment that the possibility of adverse effects on chronic kidney disease and systolic blood pressure could not be excluded in some adults that are high consumers of game (e.g. one 200 g meal per week), i.e. they could be at risk of cardiovascular effects and nephrotoxicity. Some children in average consumer groups across the EU (that did not frequently ingest wild shot game) may already be at risk of reduced IQ. Any consumption of foodstuffs with elevated lead levels, such as game shot with lead, would amplify this risk in this particularly vulnerable group. While all of these health effects, on IQ, systolic blood pressure and chronic kidney disease have associated economic costs, we have estimated only the costs of IQ reduction in children, the most vulnerable group.

#### Costs of estimated reductions in IQ in children

Several estimates exist of the number of children under eight years old in the UK at risk of incurring a one point or more reduction in IQ as a result of their current levels of exposure to ammunition-derived dietary lead from game. Green and Pain (2015) estimated this to be thousands of children in the UK (calculated to be in the range 4 000—48 000) at risk from lead exposure via gamebird meat alone. An unpublished British Association for Shooting and Conservation/Countryside Alliance (BASC/CA) game meat consumption survey estimated that 9 000 (midpoint of 5 500—12 500) children (it is unclear whether these were under eight years old or less than eight years in age so we have assumed the latter) from the shooting community consume at least one game meal per week averaged over the year (reported in LAG 2014). A human health assessment of the risks associated with consumption of game shot with lead (LAG 2015) indicates that 11 000 children (ages unspecified) from the shooting community eat at least one game meal per week. Both of the latter two estimates exclude high-level consumers of game meat outside the shooting community and refer to all types of game, but it is likely that the vast majority of it was wild game killed using lead ammunition. These estimates are for the live-quarry shooting community only and for children eating one or more game meals a week. This level of consumption generally exceeds the amount of game required to give the BMD for neurodevelopmental effects (Green and Pain 2012; Green and Pain 2015). Hence (and noting the BMD is less conservative than BMDL as described above), it seems probable that the population of children of 8 years old or younger at potential neurodevelopmental risk from ammunition-derived lead in game meat in the live-quarry shooting community in the UK may be more than 10 000.

The implication of this exposure to lead (to the BMD) has been estimated as a 1 point or more decrease in IQ in children (EFSA 2010), which can have a significant cost to society. This cost could be calculated for the EU by estimating the number of children across the EU that consume enough game to potentially have a negative impact on their IQ, and applying relevant valuations for the costs associated with that IQ reduction.

The actual amount of wild game consumed in all EU countries is not known, but can be estimated approximately for children by scaling the number of UK children exposed to high dietary levels of ammunition-derived lead by the number of hunters in other EU countries, relative to the UK. This approach is not expected to be completely accurate because we do not know how much per capita game consumption by hunters and their families varies among EU countries. The total number of hunters in the EU28 is estimated to be over 6 667 770 in 2009 (based on a survey reported by FACE 2010). Based on these data, and applying the UK hunter to child game consumption ratio (800 000 hunters and 10 000 children estimated to be at neurodevelopmental risk), around 83 000 or more children across the EU27 may be at risk of a potential reduction in IQ of 1 point.

Studies in the USA have related a 1-point reduction in IQ to a 4.5% increased risk of failure to graduate from high school and a 2% decrease in productivity in later life (Schwartz 1994; Grosse et al. 2002). In the EU, although they use different methods, two different studies have valued a reduction in 1 point in IQ (per child) based upon reviews of the literature, at around €8 000 and €10 000 (ECHA 2011; Bierkens et al. 2012). More recently, Monahan et al. (2015) estimated the discounted lifetime monetary value of the loss of one IQ point as being considerably lower at £3 297 (€3 882 in 2018 prices^7^). This corresponds to the cost of a 1 point decrease in IQ to a child across their lifetime. Using this range of values (€3 882—10 000), the consumption of lead-shot game by children within the EU today may be linked to a potential loss in IQ estimated to be worth €322 million to €830 million. This is a cost to the cohort of children 8 years old or younger. If we divide by 8 we have an annualised cost of €40 million—€104 million, i.e. the recurring (i.e. ongoing and cumulative) cost to society for every year in which use of lead shot and rates of consumption of lead in game meat food persist at current levels. Historic impacts prior to the generation considered here are not evaluated but are additional.

This calculation is conservative in several ways. Firstly, some children will eat more than one meal of game a week, with risk of a greater reduction in IQ. Secondly, recent studies suggest that high-level consumers of game may be more numerous, relative to the national number of hunters, in some other EU countries than in the UK. In the UK, with 800 000 hunters, one survey estimated that 27 000–62 000 adults eat game more than once a week and 5500–12 500 children eight years old or younger eat game once a week (cited in LAG 2014). These 32 500–74 500 people of all ages amount to only about 10% of the population of hunters. Studies conducted in other EU countries suggest that about 2–3 times the population of hunters may be potential high consumers of game. For example, in Italy, Ferri et al. (2017) surveyed 766 Italian shooters and found that an average of four servings per month (of 100–200 g game per serving) was consumed and that game mammals and birds were consumed regularly with friends and relatives in 83% and in 60% of cases, respectively. Accounting for an inventoried population of 751 876 shooters in Italy, these authors estimated that there is regular consumption of mammalian and feathered wild game in around 1.65 million and 2 million people, respectively—equating to 2.2–2.7 times the number of hunters in Italy. In Germany, Gerofke et al. (2018) found, from a representative survey conducted on game meat consumption of the German population, that about 1.5% ate large game (red Deer (*Cervus elaphus*), roe deer (*Capreolus capreolus*) and wild boar (*Sus scrofa*)) once a week or more and an additional 2.4% one to three times a month. With a population of 82.8 million (in 2017—Eurostat online), the 1.5% of at least weekly consumers of game meat represents 1.24 million people, which is over three times the population of hunters (in 2016/17—c. 384 000; DJV 2017). It therefore seems likely that in some other EU countries high-level consumers of game may be much more numerous, relative to the total number of hunters, than in the UK. Thus, our estimate of costs of IQ reduction across the EU, which is based upon UK proportions, could be too low. Game consumption could also be higher than that in the UK, relative to the number of hunters, in Sweden and France (Livsmedelsverket undated; ANSES 2018).

We have not considered the cost contribution that increased blood lead levels may potentially make to increased crime rates (e.g. see Campbell et al. 2018). Criminality has costs to the criminal justice system and to victims, including in health care, lost earnings, stolen/damaged property and loss of quality and duration of life. While there is compelling evidence linking childhood lead exposure and antisocial behaviour in childhood and later adolescence (Sampson and Winter 2018), this area is understudied and we have not attempted to monetise potential economic costs.

#### Other health costs

Reduced IQ in children is only one of the health effects associated with chronic low-level exposure to lead, as can occur through the frequent consumption of game animals shot with lead ammunition. EFSA (2010) considered that the possibility of adverse effects on chronic kidney disease and systolic blood pressure could not be excluded in adults with high levels of wild game consumption, and we have not attempted to evaluate the costs to adult health. A large scale longitudinal study from the USA has recently reported that many more adult deaths appear to be associated with low level lead exposure than previously considered. Results suggest that low-level environmental lead exposure is an important and largely overlooked risk factor for death, particularly from cardiovascular disease, in the USA (Lanphear et al. 2018). In the EU, approaching 49 million people were living with cardiovascular disease, with an estimated to cost the economy of €210 thousand million a year (Wilkins et al. 2017)—averaging €4286 per person per year. While increased systolic blood pressure in frequent consumers of game may only contribute a small proportion to this, the economic costs may nonetheless be substantial (e.g. a totally hypothetical contribution of 0.1% increase in cardiovascular disease contributed by increased lead consumption from among the 7 million hunters and c. 21 million associated game consumers would cost €120 million a year).

Also, ingestion is only one route of exposure to lead from ammunition, albeit possibly the most significant in many exposed people such as hunters and their families. Elevated blood lead levels are also associated with hunting activity per se, whether by subsistence hunting communities or target shooters (e.g. Fillion et al. 2014; Laidlaw et al. 2017). This is likely associated with inhalation of lead fume or the transfer of lead dust (e.g. when handling lead ammunition) (reviewed in Green and Pain 2015). While use of non-toxic shot would prevent exposure to lead dust (due to the abrasion of lead shot), it would not prevent exposure to lead fume resulting from the use of lead compounds in the chemical mixture of the primer.

### Costs associated with environmental impacts

Lead from ammunition is a significant and largely unregulated source of environmental contamination across Europe. Most emissions are strictly regulated and controls exist on lead levels in the ambient air, ground water, surface water, drinking water, soils, battery disposal, landfill, petrol and other sources (see AMEC 2012). As an example of the significance of ammunition emissions, in Norway in 2005, ammunition and fishing equipment (weights etc.) were considered to constitute 90% (66% and 24%, respectively) of the total of 240 tonnes of Norwegian lead emissions (Heier et al. 2009) with industrial deposits contributing only 3%.

According to industry figures, annually approximately 21 000 tonnes of lead from shotgun cartridges used in hunting is dispersed into the environment in the EU (27) with an additional 10 000–20 000 tonnes used by sports shooters (ECHA 2018a^8^ based on a variety of figures including AMEC 2012). This reflects the suggestion by the Association of European Manufacturers of Sporting Ammunition (AFEMS—as reported in AMEC 2012) that approximately half of all lead shot consumed in the EU is used for target shooting and the other half is used for hunting. Lead from bullets is additional with an estimated 350 + tonnes dispersed into the environment by hunting in 2004 (ECHA 2018a). Lead from ammunition is unevenly distributed in the environment. Highest concentrations are found where shooting occurs consistently in limited areas, e.g. at static target shooting ranges (like military ranges), moving target ranges (like clay pigeon shooting sites) and where live game are shot from static blinds (e.g. Andreotti and Borghesi 2012). Other types of live target shooting, including driven gamebird shooting, disperse lead ammunition more widely across large parts of the countryside.

Once deposited, a high proportion of lead from shot usually stays in the upper soil layers and generally breaks down slowly, with some lead being leached to the surrounding environment. In areas of high ammunition deposition, soil concentrations can be up to hundreds of times higher than in uncontaminated control sites (summarised in LAG 2015). In certain situations, some of the lead from deposited shot can be taken up by plants resulting in plant lead levels that are significantly higher than those found in plants from control soils and exceed acceptable limits for animal or human foodstuff (LAG 2015).

As long as lead ammunition continues to be used (and when it is not cleaned up), it will accumulate and associated risks to human and environmental health will increase. For example, in Finland, several thousand outdoor shooting ranges exist and they were considered one of the most common causes of soil contamination, with almost a third of them considered to have the potential to cause a risk of groundwater pollution (Sorvari et al. 2006). Soil, discharge, subsurface and groundwater lead concentrations can be high in areas of repeated ammunition deposition (e.g. Mariussen et al. 2017a; Okkenhaug et al. 2017) and put at risk soil biota, small mammals and aquatic organisms including fish (Sorvari 2007; Heier et al. 2009; Mariussen et al. 2017b).

While relatively little appears to have been done to remediate environmental lead contamination from wild game shooting, contaminated soil is treated at some firing ranges. High soil lead concentrations occur in impact berms at fixed target ranges, and more broadly across sites where there is a moving target, such as at clay pigeon shoots. Metal-contaminated soil, particularly at abandoned shooting ranges, is sometimes dealt with either by removal to approved landfill sites or treatment to stabilise the lead and reduce the amount of lead and other metals that is leachable (e.g. Kajander and Parri 2014; Mariussen et al. 2015). However, remediation can be challenging and costly, especially with shooting ranges on mires where large volumes of peat may need to be removed, the availability of disposal sites for this type of material is limited, and some mires also have high conservation value and can take decades to restore (Mariussen et al. 2017a). High levels of contamination at shooting ranges may necessitate costly cleanup and/or restrict subsequent land uses, e.g. limiting potential for agricultural use, or uses that could potentially put at risk human health, domestic stock or wildlife. Such risk could result from: elevated plant lead levels (particularly in root crops); the risk of grazing domestic or wild animals ingesting either soil or plants with high lead concentrations while feeding, or shot close to the soil surface; and risks presented by silage made from plants from shot fall-out areas, that could contain lead pellets. Health risks resulting from redeployment of shot-over land may not become apparent for some time. For example, Urrutia-Goyes et al. (2017) found a high (non-carcinogenic) health risk due to Pb pollution, with ingestion as the main exposure pathway, at an urban public park on the redeveloped site of a historic military shooting range in Athens, Greece.

There is no register of shooting clubs and ranges across Europe, and these vary in size from large establishments used on a daily basis to small parts of shooting clubs that are used only occasionally. In Finland, Kajander and Parri (2014) estimated that between 600 and 1000 shooting ranges existed. If the ratio between the proportion of hunters and shooting ranges in Finland holds across the EU, this would suggest that approximately 17 000 shooting ranges exist across the EU (using numbers of hunters from FACE 2010). Kajander and Parri (2014) produced a detailed analysis of the best available techniques for the management of environmental impact at shooting ranges. These include design features for new ranges, maintenance measures and remediation. Some of these can be very costly, but due to the variation in the types of shooting activities at ranges and the environments in which they are situated, site-specific studies are needed to identify appropriate management methods and a single best available technique cannot be identified for all situations.

Costs of clean-up will be associated with individual situations, and few estimates exist based upon a cost per tonne of lead ammunition contaminating the land. However, it was recently reported in the press in the USA (Kays 2018) that the clean-up costs of an estimated 60 tons of lead bullets (54.4 tonnes) was US $500 000 (€440 000). Extrapolating this to the 10 000–20 000 tonnes of lead gunshot used by sports shooters in the EU annually would suggest that, were all lead contamination to be mitigated, annual costs would be in the region of 92 million to 184 million $US (€81–162 million). This estimate is for bullets and clean-up of gunshot is likely to require that larger areas be treated as gunshot are more dispersed than bullets. Furthermore, there is a large margin of error associated with this estimate as it is based on just one recent decontamination example, but it gives a very broad indication of hypothetical annual costs. While it would not be practical or economically feasible to clean-up the 21 000 tonnes of shot used annually for hunting, it seems reasonable to assume that at least a similar cost would likely be required to reduce risks in the most contaminated areas, such as regular blinds.

### Other costs

#### Surveillance and research

Surveillance and research on the impacts of lead poisoning (including monitoring the efficacy of regulations where they occur) is time consuming and costly. The evidence which has then driven policy on this issue has come mainly from scientists from universities and the conservation NGO sector. Scientists have been required to demonstrate that lead from ammunition presents unacceptable risks to wildlife, human health and the environment rather than for the shooting users (the polluters) to demonstrate its safety. In order to develop an indicative cost of this research, peer-reviewed studies conducted over a 5-year period (Feb 2013–Jan 2018) in Europe were identified using Web of Science (Table 4). The estimated research cost over this period was €2.45 million. This does not include studies that have been published but not peer-reviewed, including many government reports and risk analyses conducted by government agencies. These can be extremely costly, for example in the UK, the Lead Ammunition Group (LAG) was set up to advise the UK Government’s Department for the Environment, Food and Rural Affairs (DEFRA) and the Food Standards Agency (FSA) on the risks of lead ammunition to wildlife and human health, and potential mitigation measures. The LAG conducted a series of risk assessments (LAG 2015) running to > 400 pages and held 18 meetings between February 2013 and January 2018. This process alone (funded by the individual members and their supporting organisations rather than government) is likely to have cost in the region of £200 000–£300 000 (€236 000–€354 000) in staff time over a 5 year period. In addition, several other research reports were conducted by or commissioned by UK Statutory agencies over this period (i.e. the Food Standards Agency and FSA Scotland). Human health risk assessments were also conducted in a variety of other European countries, including Spain, France, Germany, Norway and Sweden (AESAN 2012; VKM 2013; SNFA 2014; ANSES 2018; Gerofke et al. 2018). It would therefore not be unreasonable to suggest that the total annual cost of research into this issue, including university, NGO and government scientists, is likely to be in the region of €1 million or more annually. This does not include the substantial amount of work conducted by the European Chemicals Agency (ECHA) in the preparation of a dossier for restriction proposal for the use of gunshot over wetlands, nor the many individuals and organisations that have contributed to this process.

**Table 4:** Estimated costs of research over a 5-year period (Feb 2013–Jan 2018) related to the impacts lead ammunition on humans, domestic and wild animals and the environment in Europe

Type of study	Number of studies (Feb 2013–Jan 2018)	Indicative cost (€) per type of study	Total cost (€)
Desk-based studies	5	14 000	70 000
Lab/fieldwork-based studies	49	44 000	2 156 000
Large studies with metadata analysis	4	57 000	228 000
Total	55	–	2 454 000

1. The total number of studies is limited to just published peer reviewed European studies carried out over the last 5 years. Studies have been gathered from Web of Science (23.08.18) and limited to research on (i) wildlife populations, (ii) domestic animals, (iii) human health, and (iv) environmental contamination. Search terms used were: lead and shot and bird; lead and bullet and bird; lead and ammunition and bird; lead and ammunition and human; blood and lead and domestic/livestock; blood and lead and game; lead and ammunition and pollution. It also excludes research on non-toxic alternatives to lead shots (i.e. focuses on the problem rather than studies on the solution)

2. The indicative cost per type of study (2016 prices) was determined using expert judgement by calculating the number of days required per type of resource required (e.g. fieldworkers, technician, research associates, senior researchers, veterinarians/medics), lab equipment and lab analysis required and a standard full cost recovery university overhead factor. The results have been verified through informal consultation with those who carry out such fieldwork and lab analysis

#### Enforcement

At present, legislation regarding the use of lead ammunition across the EU and Europe is variable. With respect to gunshot, a few EU member states have introduced legislation banning the use of lead gunshot (irrespective of species shot or habitat) across all or much of their territory (Belgium, Denmark, The Netherlands, Croatia), five have no legislation (Greece, Ireland, Poland, Romania, Slovenia) while the remaining member states have partial restrictions, e.g. for shooting certain species and/or in certain places (ECHA 2017a). Regulations should be followed in those countries that have them, but enforcement is variable. For example, in the UK where there are partial restrictions, there is little if any statutory enforcement of the regulations and very low compliance (c. 30%—Cromie et al. 2015). Were enforcement to be effective under such situations of partial regulation it would be very costly. This is a cost that should currently be incurred by governments, but is not (at least in the UK) due to a lack of enforcement and ineffectiveness of current partial regulations. A total ban on all use of lead gunshot is far simpler in terms of practicality and enforcement as acknowledged by ECHA (2018b). A total ban would be simple to police by existing enforcement organisations, and responsibility for compliance would sit with the producers and retailers of lead gunshot rather than the individual shooter. Enforcement costs would likely be far lower for a total ban compared to partial restrictions.

#### Collision

Another area of cost not previously considered is that of increased risk of collision of large birds, such as swans, with infrastructure like power lines which has been found to be related to elevated blood lead level (Kelly and Kelly 2005; Ecke et al. 2017); this is probably related to the disorientation and physical impediment created by sublethal lead levels. Associated economic impacts result from interruptions of power and damage to power lines (and potentially to road traffic). Lack of coordination resulting from lead poisoning was also suspected when an Imperial Eagle (*Aquila heliaca*) crashed into a car in Hungary in 2017 (Pannon Eagle 2018). Bird strikes with aircraft present an ongoing safety and economic risk (Pfeiffer et al. 2018). While no data are available, it seems probable that effects of sublethal lead poisoning on flight behaviour might increase the likelihood of aircraft strikes. The potential effects and costs of such strikes would be greatest for large-bodied birds such as swans, geese and eagles.

#### Food production

Contamination of land by lead from ammunition occasionally results in pollution issues for domestic animals or food production. For example, incidents of lead poisoning from ingested lead gunshot (deposited by target shooting) occasionally occur in small numbers of domestic poultry and cattle in the UK causing suffering and mortality (Payne et al. 2013; APHA 2016): this has sometimes created potential food safety incidents, illustrated by a supermarket recall of eggs from chickens that had ingested lead shot (BBC 2008). In Italy, a police operation in 2016 reportedly seized about 3000 packs of meat sauce and sauce based on game meat, due to the detection of lead levels that exceeded legally permissible limits (Piuweb 2016). A brand of sea salt produced at a salt pan in France and distributed was recalled from supermarkets due to elevated lead concentrations, apparently caused by contamination from lead ammunition (Colin 2018). While such cases of food contamination are reported relatively infrequently, they can have a serious economic impact for the farmers and food producers and distributors concerned.

The use of lead ammunition also results in considerable loss of otherwise useable meat due to the need to remove and discard meat within a large radius of the wound canal of bullets in large game animals. Fragments from bullets and elevated tissue lead concentrations have been found as far as 20–30 cm from the wound canal so considerable meat loss is associated with attempting to eliminate lead fragments (e.g. VKM 2013). Several food safety agencies recommend discarding meat in proximity to the wound canal (SNFA 2014) including a radius of 30 cm from the bullet tract (Knutsen et al. 2015). In Norway, efforts to avoid lead in venison by discarding meat close to wound channels causes the discard of 200 tonnes of contaminated meat annually,^9^ representing an economic loss equivalent to €3 million (Kanstrup et al. 2018).

#### Risk to dogs

It is common practice for hunters of large game animals to leave offal and sometimes trimmings of meat from around the wound canal in nature, and sometimes trimmings from the wound canal are fed to dogs (e.g. VKM 2013). Chronic exposure to lead through feeding wound trimmings to dogs presents a risk of lead poisoning (VKM 2013; Høgåsen et al. 2016), with associated welfare costs and costs to the animal’s owners.

## Discussion

ECHA (2017a) estimated the total annual societal costs of restricting the use of lead shot over wetlands (including peatlands) in the EU to be €35–61 million. This takes account of the costs to hunters (including costs for necessary testing, technical adaptations to shotguns, premature replacement of shotguns, and the incremental cost of more expensive alternative ammunition) and the share of this cost that goes either as tax revenue to governments or as mark-ups to retailers and manufacturers of shotguns and ammunition. ECHA used a figure of total societal benefits of > €105 million comprising the avoided opportunity cost associated with the annual mortality in the EU of approximately 700 000 wildfowl from 16 wetland bird species known to ingest lead shot (Andreotti et al. 2018). None of the other societal use, non-use or existence benefits (e.g. mortality of scavengers and predators, human health impacts, impacts on leisure activities, protection of ecosystem services and rare bird species) were quantified.

The proposed restriction covers wetlands, and ECHA (2017a) used the Ramsar definition of wetlands which includes peatlands. They assumed that 8% of shooting was in the narrower definition of wetlands (i.e. largely wetlands with open water where wildfowl shooting takes place) and that the collateral impact occurring due to the wider wetland definition affected the 53% of hunting by shooters of ‘fowl-like’ birds (e.g. grouse, partridges, quail, pheasant, dove and pigeons) that could occur over peatland. ECHA acknowledged that it is possible that the numbers of hunters over peatland may be lower than this, and therefore their estimate of costs to hunters may be an overestimate. However, as ECHA found costs of €35–61 million for 61% of hunters (using shotguns), we can broadly assume a cost of €57–100 million for all hunters who use shotguns, although this may be higher or lower. This would not include costs of restricting lead ammunition to sports (target) shooters and to large game hunters using rifles, or target shooters using bullets. However, it is considered that steel shot types available could be readily used in the types of guns used, and target shooting practiced at, the Olympics (Thomas and Guitart 2013) and by extension in most target shooting clubs.

Some non-lead alternatives like steel shot may over time become cheaper than equivalent lead shot, hence transition to non-lead includes the potential for reducing hunter’s annual [running] cost (Kanstrup and Thomas 2019). The transition to non-lead bullets would also incur a cost, but volumes are low compared with lead shot (AMEC 2012) and alternatives are available in the EU and already widely used in some places, e.g. several German States have regulations requiring the use of non-lead bullets (Thomas et al. 2016) and Forest Enterprise England wildlife rangers transitioned to using lead-free bullets for killing deer (FEE 2017). While we have not attempted to estimate the costs of complete transition to non-toxic ammunition in this paper, these factors suggest that it is unlikely to be much more than double the €57–100 million estimated for all hunters that use shotguns.

A range of benefits of banning lead ammunition, relating to avoiding costs that its use currently imposes on society, are identified. For the EU, minimum replacement (€133–565 million) and treatment (€28 million) costs for a limited selection of bird species known to die of lead poisoning are estimated at, on average, around €377 million annually. An extrapolation of a WTP study for avoided lead-poisoning losses of wildfowl alone gave an indicative value of c. €2.2 thousand million per year. Uncertainty in extrapolating WTP values from Scotland across EU member states, and the limited number of species for which data enabled replacement and treatment cost estimates to be made suggest an annual cost of those birds lost to lead poisoning of at least several hundreds of millions and possibly several thousand million euros. Costs to human health are likely to be associated primarily with reduced IQ in children and increased cardiovascular and chronic kidney disease in adults. Minimum annual costs of reduced IQ in children are estimated at €40 million–€104 million but these could be higher, possibly substantially higher. Health costs in adults have not been estimated but could be of a similar order of magnitude taking account of the potential numbers of high consumers of game and the costs of healthcare. The costs of environmental clean-up of shooting ranges have only been estimated in the broadest terms but, based on clean-up of the tonnage of lead estimated to be used at shooting ranges, could hypothetically be €81–162 million for shooting ranges and it seems reasonable to suspect may be similar or higher at heavily contaminated sites of wild game shooting such as hunting blinds.

Table 5 summarises estimated additive costs to wildlife, human health and the environment and lists additional costs that would also be additive but that we have not been able to estimate. Those un-estimated costs likely to be most substantially are human health costs of frequent exposure to lead from game in adults (chronic kidney disease and systolic blood pressure) and replacement and treatment costs of those bird species known to be affected by lead poisoning but for which insufficient data were available to make estimates. Estimates of numbers of people that frequently consume game in some EU countries also suggest that our estimate of the costs of reduced IQ in children associated with frequent game consumption may be low. Our estimates of several of the costs of continued use of lead ammunition have involved a large number of assumptions, as laid out in Tables 1, 2, 3, 4 and 5 and the accompanying text. However, except in the hypothetical case of environmental clean-up, our estimates have tended to be conservative, and may have underestimated costs as described. Nonetheless, the margins of error are likely to be large and the estimates should be considered to give an indication of the likely magnitude of costs rather than a precise evaluation. For additive direct costs, we estimate a minimum annual cost across the EU and Europe of c. €383–960 million and €444 million to €1.3 thousand million per year, respectively. Using a WTP approach, the value that society places on being able to avoid these losses is likely to be far higher, and was estimated to be €2.2 thousand million per year for wildfowl in the EU alone. The combined value that society would place on being able to avoid the combined wildlife, human health and environmental costs of continued use of lead ammunition would be far greater. Regardless of the methods used, our estimated costs of the continued use of lead ammunition across the EU are many times, and possibly an order of magnitude greater than the estimated annual total societal costs of switching to non-toxic alternative ammunition types.

**Table 5:** Summary of selected additive cost estimates of continued use of lead ammunition across the EU and Europe

Cost Area	Description	Annual cost (€) EU	Annual cost (€) Europe	References
Wildlife				
Replacement costs—direct mortality	Replacement of 700 000 wildfowl (EU) or 1 million (Europe) of 16 species	€105 million	€142 million	Andreotti et al. (2017)
	Replacement of 4 species of raptor in the EU (530–691 individuals) and Europe (765–1139 individuals)	€25–457 million	€37–750 million	This paper, Table 2
	Replacement of 232 402 released pheasants and red-legged partridges in the UK	Costs for UK only—€3.4 million Not estimated for EU	Not estimated but > €3.4 million	This paper, Table 1
	Replacement of an additional 11 wildfowl species; 12 raptor species, 11 other waterbird and wading species and 2 terrestrial gamebirds known to suffer lead poisoning but for which insufficient information was available to estimate replacement costs	Not estimated	Not estimated	Andreotti et al. (2018), Pain et al. (2015), (2019)
Replacement costs—indirect mortality	Birds that die as a result of sublethal lead poisoning increasing susceptibility to disease and accidents	Not estimated	Not estimated	e.g. Kelly and Kelly (2005), Ecke et al. (2017)
Treatment costs	Costs for treating 1% of 700 000 lead-poisoned wildfowl in the EU and 1 million in Europe, plus 1% of an additional 2.1 (EU) and 3 million (Europe) that suffer sublethal welfare effects	€28 million	€40 million	This paper; ECHA (2017b)
	Costs of treating raptors and terrestrial birds that suffer lead poisoning	Not estimated	Not estimated	
People				
Reduced IQ in children	Minimum annual costs of risk of reduced IQ in children in the EU that frequently consume game shot with lead. Surveys from other countries suggest that this may be an underestimate, possibly by an order of magnitude	€40–104 million	Not estimated but > €40–104 million	This paper
Increased incidence of CKD and SBP in adults		Not estimated	Not estimated	
Environment				
Clean-up	Ammunition at shooting ranges—broad estimate	€81–162 million	Not estimated but > €81–162 million	This paper
Clean-up	Ammunition at hunting blinds with greatest contamination—broad estimate	c. €100 million	Not estimated but > c. €100 million	This paper
Other costs				
Research	Investigating lead poisoning; monitoring and surveillance	€ 1 million	Not estimated but > € 1 million	This paper, Table 4
Advocacy		Not estimated	Not estimated	
Enforcement		Not estimated	Not estimated	
Collision	Collision of poisoned birds with power lines, other infrastructure and vehicles due to weakened state	Not estimated	Not estimated	Kelly and Kelly (2005), Pannon Eagle (2018)
Food production	Poisoning of poultry and livestock exposed to lead shot or feed contaminated with lead shot; other food products (e.g. salt) contaminated with shot; wastage of meat around the wound channel of large mammals killed with lead bullets	Not estimated	Not estimated	Payne et al. (2013), APHA (2016), Colin (2018)
Health of domestic dogs	Risks to dogs fed trimmings from shot game animals	Not estimated	Not estimated	VKM (2013), Høgåsen et al. (2016)
Total		€383 million–€960 million	€444 million–€1.30 thousand million	

## Footnotes

1 This process was still underway February 2019.

2 Based on €60 for vet examination, €315 for anaesthetic and X-ray, 2 × €126 for blood test, €315 for 5 day hospitalisation and €88 for chelation therapy.

3 Throughout the paper, figures have been presented in £ when this was the currency of the original publication cited, with a € conversion given using an exchange rate of £1 = €1.18 (November 2018).

4 Throughout the paper, figures have been presented in $ when this was the currency of the original publication cited, with a € conversion given using an exchange rate US$1 = €0.88 (November 2018).

5 WTP values were converted from 2001 GBP to 2017 Euros using the 2001 to 2017 Consumer price index https://www.ons.gov.uk/economy/inflationandpriceindices/timeseries/d7g7/mm23 and the Bank of England’s exchange rate (Bank of England, 2018) https://www.bankofengland.co.uk/boeapps/database/Rates.asp?Travel=NIxASx&into=EUR (for 21/11/2018) and the ratio between EU and UK average household disposable income per capita.

6 Eurostat number of private households http://appsso.eurostat.ec.europa.eu/nui/show.do?dataset=lfst_hhnhtych&lang=en

7 Converted from 2015 GBP to 2017 Euros using money GDP deflators at Market Prices (HM Government, 2018) https://www.gov.uk/government/statistics/gdp-deflators-at-market-prices-and-money-gdp-october-2018-budget-2018) and the Bank of England’s exchange rate for 21/11/2018 (2018 https://www.bankofengland.co.uk/boeapps/database/Rates.asp?Travel=NIxASx&into=EUR).

8 Other estimates of annual releases of lead gunshot indicate the tonnage is probably significantly higher (see ECHA 2018a).

9 http://www.hegnar.no/Nyheter/Naeringsliv/2016/09/Maa-kaste-200-tonn-viltkjoett.
